# Supplementary material for: Exploiting metabolic acidosis in solid cancers using a tumor-agnostic pH-activatable nanoprobe for fluorescence-guided surgery
Source: Nat Commun. 2020 Jun 26;11:3257. doi: 10.1038/s41467-020-16814-4 (PMC7320194; doi:10.1038/s41467-020-16814-4)
Supplement: Supplementary file 1 — Supplementary Information [file 41467_2020_16814_MOESM1_ESM.pdf]

Supplementary data

Exploiting metabolic acidosis in solid cancers using a tumor-agnostic pH-activatable nanoprobe for fluorescence-guided surgery

Voskuil et al.

Supplementary Figures

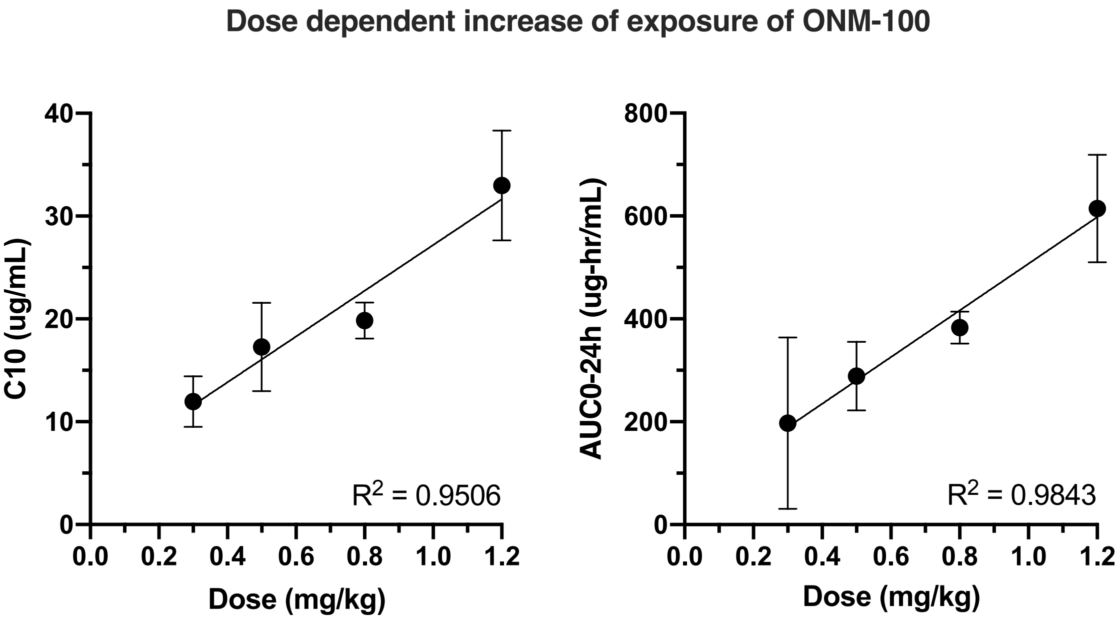

Supplementary Figure 1 | Dose dependent pharmacokinetics

There is a dose-dependent correlation between the mean plasma concentration of ONM-100 ten minutes after administration and the mean area under the curve in the first 24 hours with a R^2 of 0.95 and 0.98 respectively. Dots represent mean values per cohort, error bars standard deviation. N=3 for 0.3, 0.5 and 0.8mg per kg cohorts, N=18 for 1.2mg per kg cohort. Abbreviations: C10m: Plasma concentration at 10 minutes; AUC0-24h: Area under the curve in the first 24 hours; ug: micrograms; mL: milliliters; hr: hour. Source data are provided as a Source Data file.

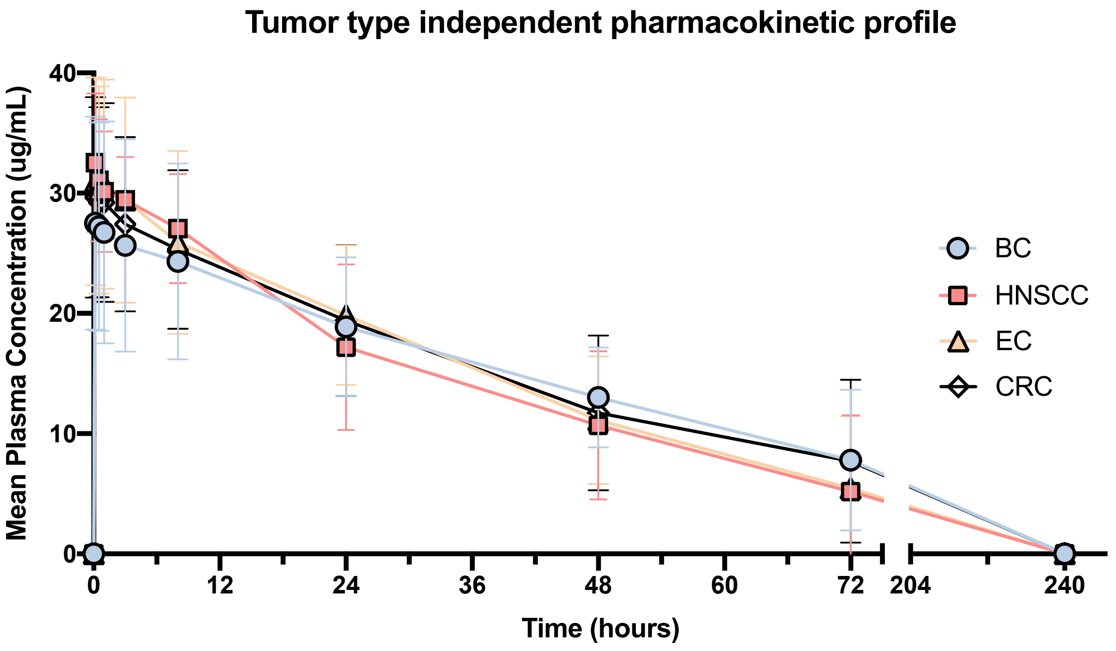

Supplementary Figure 2 | Pharmacokinetic comparisons of different tumor types

There were no apparent pharmacokinetic differences between the subjects who received 1.2 mg per kg, based on tumor type. The mean terminal-phase half-life in the 1.2 mg per kg cohort was 44.5 with a standard deviation of 15.8 hours. Dots represent mean values per tumor type (N=3-13, depending on number of patients), error bars standard deviation. Abbreviations: BC: Breast Cancer; HNSCC: Head and Neck Squamous Cell Cancer; EC: Esophageal Cancer; CRC: Colorectal Cancer. Source data are provided as a Source Data file

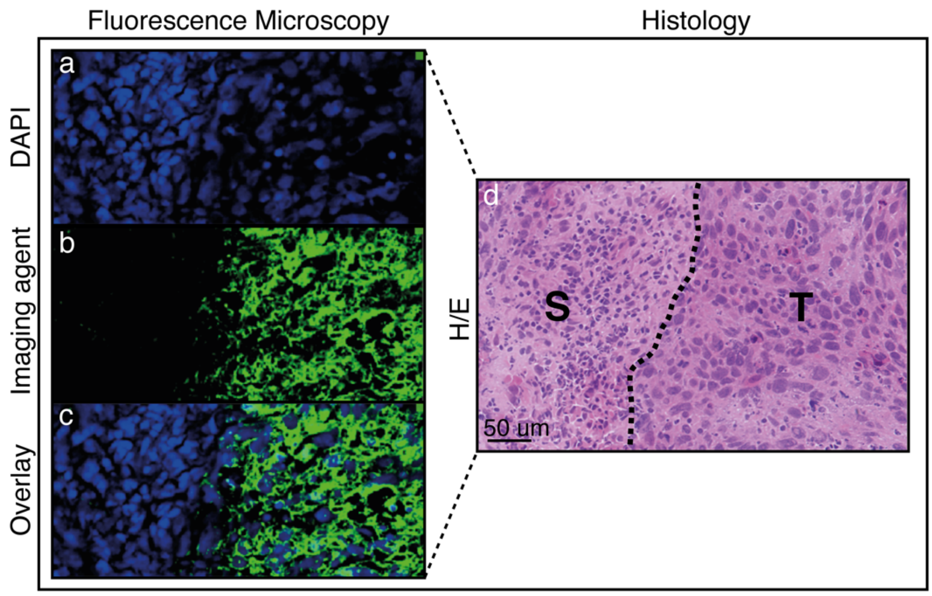

Supplementary Figure 3 | Fluorescence microscopy to confirm tumor-specific activation of ONM-100

Fluorescence microscopy was performed ex vivo after spraying ONM-100 onto tissue sections of a freshly frozen HNSCC specimen directly after excision. DAPI was applied for nuclear staining (blue) (a) and ONM-100 (green) for fluorescence visualization (b). A sharp delineation of fluorescence between the tumor and stromal tissue (c) was observed and correlated with corresponding histopathology tissue sections stained with hematotoxyline and eosin (d). Abbreviations: HNSSC: Head and Neck Squamous Cell Carcinoma; T: Tumor; S: Stroma; H/E: Hematoxylin Eosin; DAPI: 4',6-diamidino-2-phenylindole. Experiment has been repeated three times.

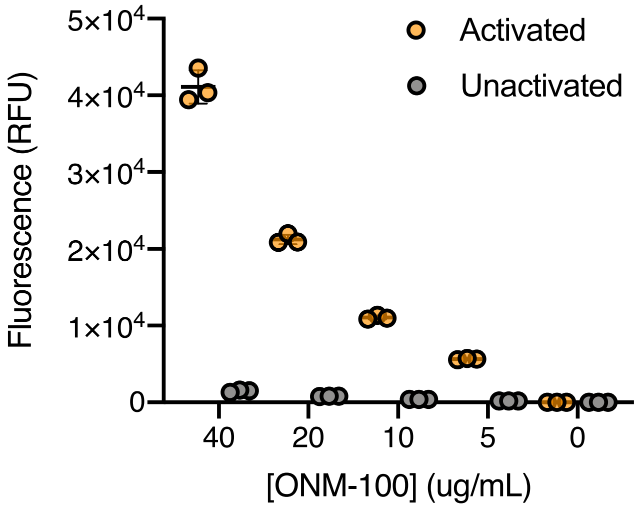

Supplementary Figure 4 | pH-dependent activation of ONM-100 in human plasma

Increasing amounts of ONM-100 were added to human plasma which did not show an increase in fluorescence. When the experiment was repeated after the addition of HCl to supply protons to the plasma, there was an increase in fluorescence with the addition of increasing amounts of intact ONM-100 suggesting that acidosis was activating the ONM-100 and thus the fluorescence in a dose-dependent manner. Dots represent individual data points. Error bars represent standard deviation. Abbreviations: RFU: Relative Fluorescence Units.

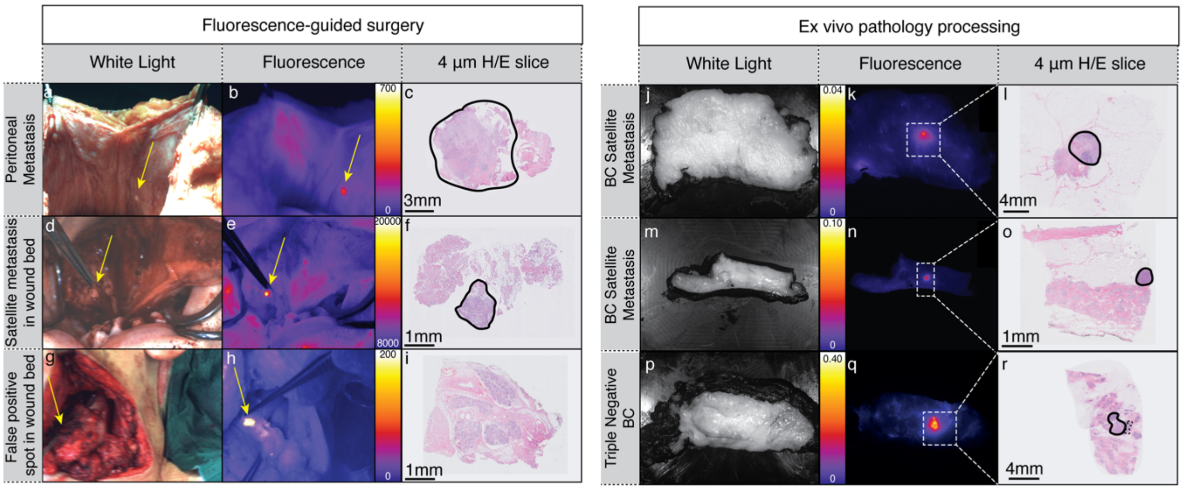

Supplementary Figure 5 | Clinically Relevant images

Intraoperatively detected Peritoneal Metastasis (PM) (a-c). An additional tumor lesion detected in the surgical cavity after a Head and Neck Squamous Cell Carcinoma (HNSCC) resection of the mandible (d-f). A false positive fluorescent lesion from salivary gland tissue (g-i). Additional satellite metastases of the primary tumor lesion were detected in two BC subjects and confirmed by final histopathological examination (j-o). An additional primary tumor lesion was detected on a fresh tissue slice from a BC subject showing triple negative breast cancer which was not detected before and during surgery (p-r). The tumor is delineated as a solid black line in the H/E slices (c,f,l,o,r). The false positive contained no viable tumor tissue (i). Abbreviations: H/E: Hematoxylin Eosin; BC: Breast Cancer.

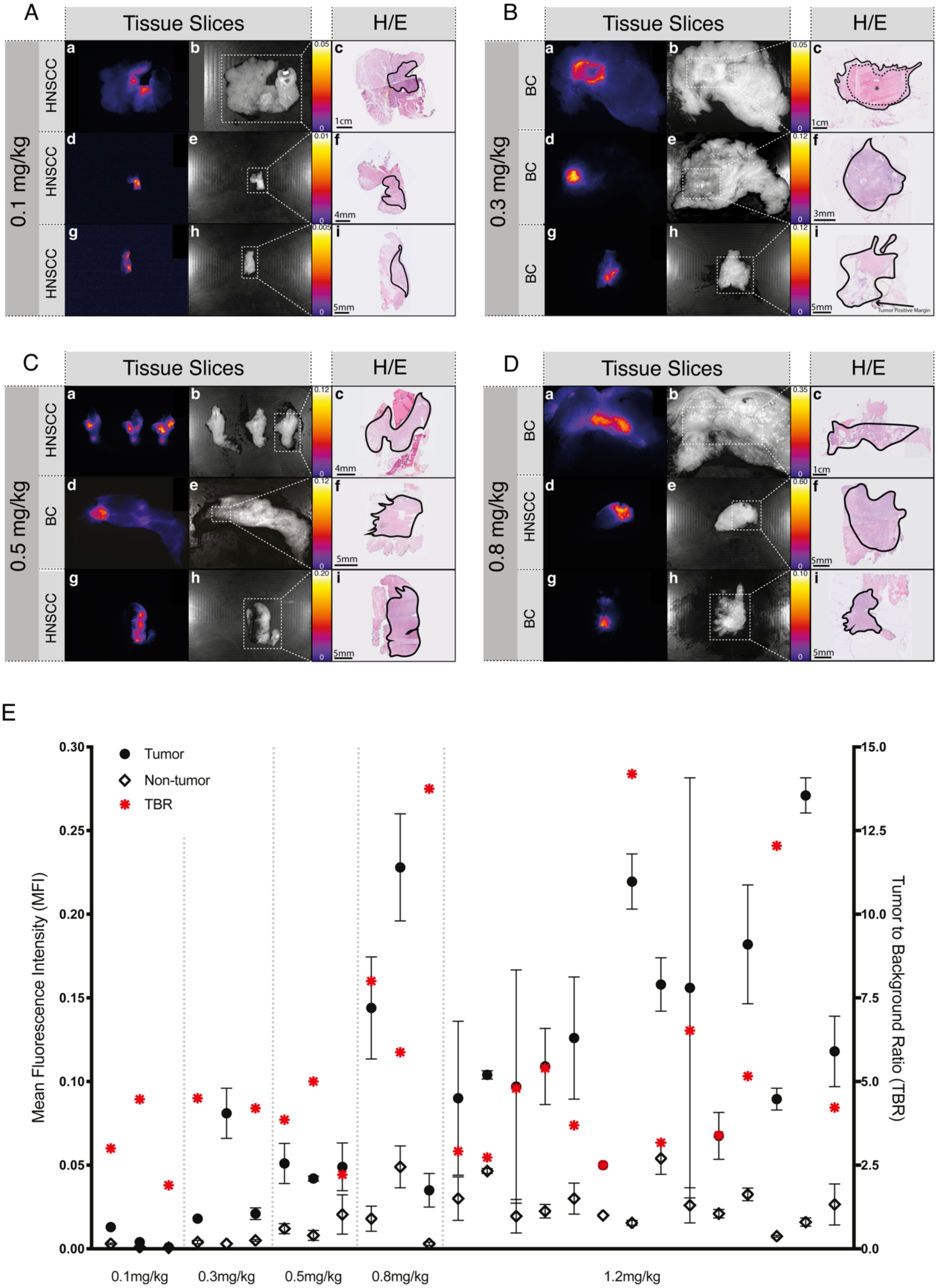
A
Tissue Slices
H/E
0.1 mg/kg
HNSCC
HNSCC
HNSCC
1cm
4mm
5mm
B
Tissue Slices
H/E
0.3 mg/kg
BC
BC
BC
1cm
3mm
5mm
Tumor Positive Margin
C
Tissue Slices
H/E
0.5 mg/kg
HNSCC
BC
HNSCC
4mm
5mm
5mm
D
Tissue Slices
H/E
0.8 mg/kg
BC
HNSCC
BC
1cm
5mm
5mm
E
Tumor
Non-tumor
TBR
Mean Fluorescence Intensity (MFI)
Tumor to Background Ratio (TBR)
0.00
0.05
0.10
0.15
0.20
0.25
0.30
0.0
2.5
5.0
7.5
10.0
12.5
15.0
0.1mg/kg
0.3mg/kg
0.5mg/kg
0.8mg/kg
1.2mg/kg

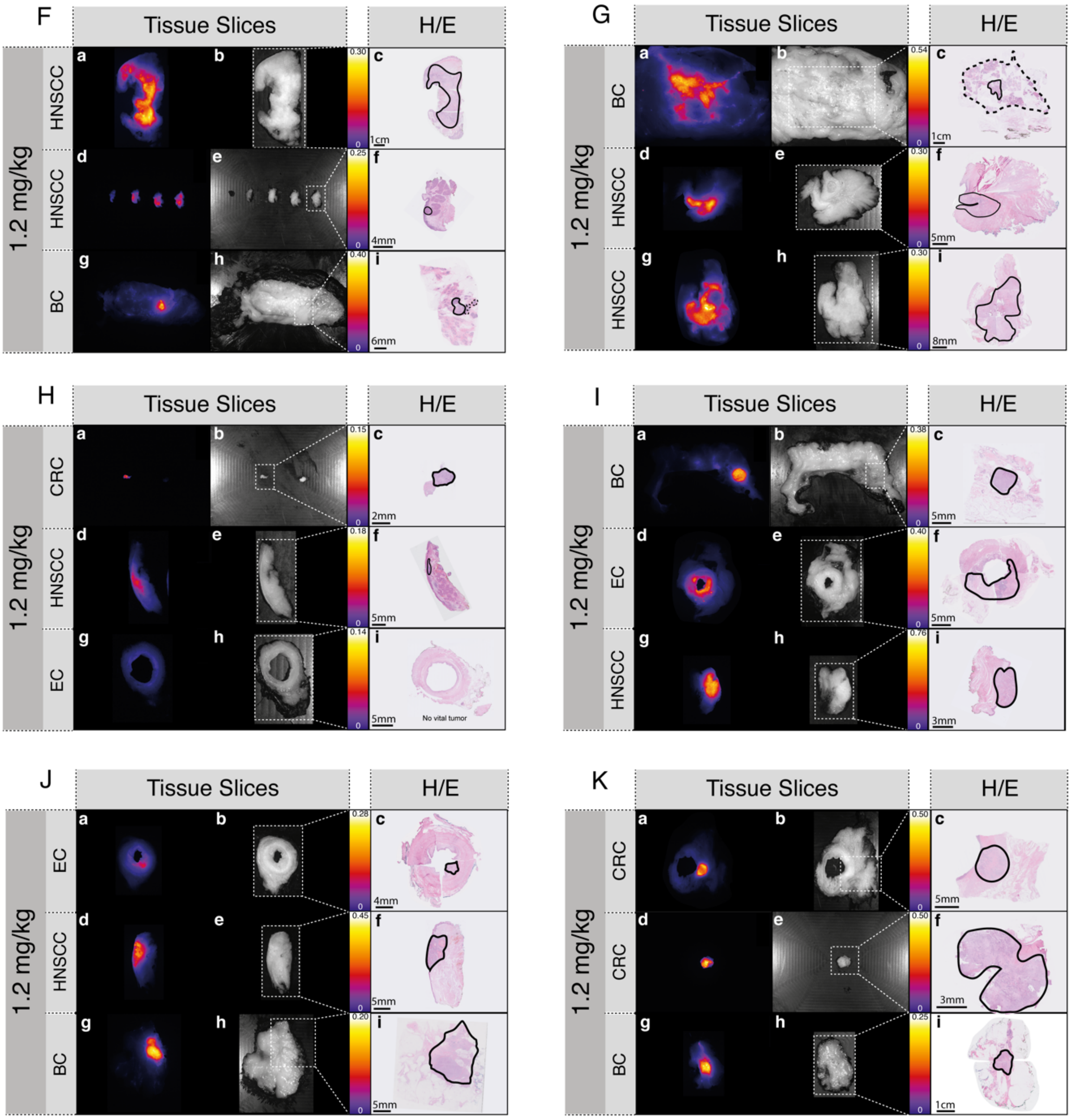

Supplementary Figure 6 | Representative images per subject

Representative sequences of white light and fluorescent images of tissue slices with the corresponding H/E 4um slice per subject. **A**: 0.1mg per kg dose cohort. HNSCC, subject 1 (a-c). HNSCC, subject 2 (d-f). HNSCC, subject 3 (g-i); the solid line on the H/E slice represents the tumor. **B**: 0.3mg per kg dose cohort. BC, subject 4. The viable tumor is between the solid and dashed line on the H/E slice. The dashed line represents the necrotic area on a H/E slice (a-c). BC, subject 5 (d-f). BC, subject 6. The arrow indicates a positive surgical margin (g-i). The solid

line on the H/E slice represents the tumor. **C**: 0.5mg per kg dose cohort. HNSCC, subject 7 (a-c). BC, subject 8 (d-f). HNSCC, subject 9 (g-i). The solid line on the H/E slice represents the tumor. **D**: 0.8mg per kg dose cohort. BC, subject 10 (a-c). HNSCC, subject 11 (d-f). BC, subject 12 (g-i). The solid line on the H/E slice represents the tumor. **E**: The MFI on all the subjects' tumor tissue slices was higher compared to non-tumor tissue. Dots and asterisks represent median values (N≥3-8, depending on availability of tissue slides), error bars represent interquartile ranges. **F**: 1.2mg per kg dose cohort. HNSCC, subject 13 (a-c). HNSCC, subject 14 (d-f). BC, subject 15 (g-i). The solid line on the H/E slice represents a tumor. **G**: 1.2mg per kg dose cohort. BC, subject 16 (a-c). HNSCC, subject 17 (d-f). HNSCC, subject 18 (g-l). The solid line on the H/E slice represents a tumor. The dashed line on the H/E slice represents a Ductal Carcinoma in Situ. **H**: 1.2mg per kg dose cohort. CRC subject 19 (a-c). HNSCC, subject 20 (d-f). No vital tumor present after neo-adjuvant treatment of EC therefore complete response, subject 21 (g-i). The solid line on the H/E slice represents a tumor. **I**: 1.2mg per kg dose cohort. BC, subject 22 (a-c). EC, subject 23 (d-f). HNSCC, subject 24 (g-l). The solid line on the H/E slice represents a tumor. **J**: 1.2mg per kg dose cohort. EC, subject 25 (a-c). HNSCC, subject 26 (d-f). BC, subject 27 (g-i). The solid line on the H/E slice represents a tumor. **K**: 1.2mg per kg dose cohort. CRC subject 28 (a-c). CRC, subject 29 (d-f). BC, subject 30 (g-i). The solid line on the H/E slice represents a tumor. Abbreviations: H/E: Hematoxylin Eosin, HNSCC: Head and Neck Squamous Cell Carcinoma, BC: Breast Cancer, EC: Esophageal Cancer, CRC: Colorectal Cancer. TBR: Tumor to Background Ratio. MFI: Mean Fluorescence Intensity, IQR: Interquartile range.

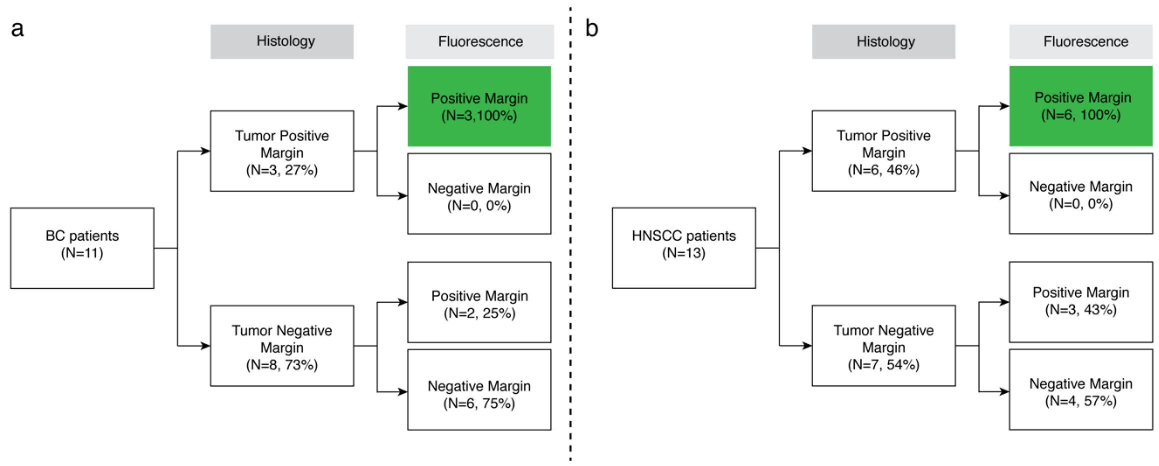

Supplementary Figure 7 | Correlating fluorescence surgical margin assessment with final histopathology results

Intraoperative assessment of the surgical margin during fluorescence-guided surgery can be done either by intraoperative fluorescence imaging of the surgical cavity or fluorescence imaging of the excised specimen at the *‘Back-Table’*. The final histopathology is correlated with the fluorescence images of breast cancer subjects (a) and head and neck squamous cell carcinoma subjects (b). Abbreviations: BC: Breast Cancer, HNSCC: Head and Neck Squamous Cell Carcinoma.

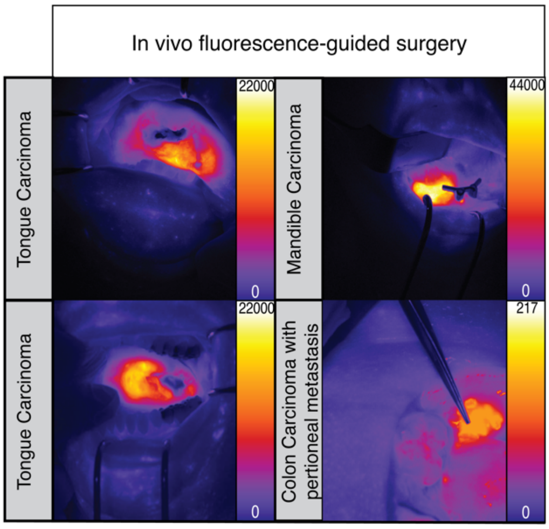

Supplementary Figure 8 | In vivo imaging using ONM-100 fluorescence

Representative examples of in vivo imaging data using ONM-100 fluorescence. A large tongue carcinoma with a central necrotic ulcer was in vivo visualized using ONM-100 (a). A cancer located at the right mandible / floor of mouth was in vivo visualized using ONM-100 (b). A large tongue carcinoma with a central necrotic ulcer was in vivo visualized using ONM-100 (c). A colorectal carcinoma with extensive peritoneal metastases was in vivo visualized using ONM-100 (d).

Supplementary Tables

Patient ID	Dose (mg per kg)	Weight (kg)	Absolute tracer dose (mg)	Tumor Type	Stage (TNM)	Max. tumor size
ON1101	0.3	83.5	25.05	BC	pT2N3aM1	3.0cm
ON1102	0.3	113	33.9	BC	pT1cN0	1.1cm
ON1103	0.3	71	21.3	BC	pT2N0	2.5cm
ON1104	0.5	92	46	HNSCC	pT4N1m0	3.0cm
ON1105	0.5	52	26	BC	pT2N0	2.8cm
ON1106	0.5	119.2	59.6	HNSCC	pT3N2bM0	5.0cm
ON1107	0.8	93.7	74.96	BC	PT3N3a	6.5cm
ON1108	0.8	93	74.4	HNSCC	pT3N0Mx	3.2cm
ON1109	0.8	73	58.4	BC	pT1cN0	1.1cm
ON1110	0.1	64	6.4	HNSCC	pT3N0mx	2.3cm
ON1111	0.1	53	5.3	HNSCC	pT1N0Mx	2.0cm
ON1112	0.1	69	6.9	HNSCC	pT2N0	1.4cm
ON1113	1.2	65	78	HNSCC	pT3N2bMx	4.0cm
ON1114	1.2	64	76.8	HNSCC	PT1N1Mx	2mm
ON1115	1.2	64	76.8	BC	pT1bN0Mx (2 primary tumors)	0.6cm
ON1116	1.2	72	86.4	BC	pT1cN0	1.1cm
ON1118	1.2	72	86.4	HNSCC	pT2N0	2.4cm
ON1119	1.2	89	106.8	HNSCC	pT4N0	3.8cm
ON1120	1.2	94	112.8	PC	n/a	n/a
ON1121	1.2	79	94.8	HNSCC	pT1N0	5mm
ON1122	1.2	78	93.6	EC	ypT0N0	n/a
ON1123	1.2	60	72	BC	pT1cN0	1.2cm
ON1124	1.2	96	115.2	EC	ypT3N1	5cm
ON1125	1.2	85	102	HNSCC	pT1N0	1.7cm
ON1126	1.2	96	115.2	EC	ypT2N2	5cm
ON1127	1.2	46.5	55.8	HNSCC	pT2N0	1.5cm
ON1128	1.2	87	104.4	BC + OC	pT3N0M0 + OC: no tumor	1.5 cm
ON1129	1.2	110	132	CRC + RC	ypT2N0 + pT3aN0	CRC: 1.5cm RC: 12cm
ON1130	1.2	83	99.6	CRC	pT4N1M1	Not applicable
ON1151	1.2	81	97.2	BC	pT1b	1cm

Supplementary Table 1 | Patient characteristics

Patient characteristics. Abbreviations: TNM; Tumor Nodal Metastasis tumor classification. BC: Breast cancer, HNSCC: Head and Neck squamous cell carcinoma; PC: Peritoneal metastasis; EC: Esophageal cancer; OC: Ovarian Cancer; RC: Rectal Cancer.

Patient	Tumor type	Margin status	Cavity fluo-rescence	Biopsy performed	Specimen margin fluo-rescence	Correlation
1	BC	Tumor-negative	no		no	TN
2	BC	Tumor-negative	no		no	TN
3	BC	Tumor-positive	no		yes	TP
4	HNSCC	Tumor-negative	no		no	TN
5	BC	Tumor-negative	no		no	TN
6	HNSCC	Tumor-positive	yes	*yes*	yes	TP
7	BC	Tumor-negative	no		no	TN
8	HNSCC	Tumor-negative	yes	*yes*	yes	FP
9	BC	Tumor-positive	yes		yes	TP
10	HNSCC	Tumor-positive	no		yes	TP
11	HNSCC	Tumor-positive	*n/a*		yes	TP
12	HNSCC	Tumor-negative	no		no	TN
13	HNSCC	Tumor-positive	yes	*yes*	yes	TP
14	HNSCC	Tumor-negative	no		yes	FP
15	BC	Tumor-negative	no		no	TN
16	BC	Tumor-negative	no		no	TN
17	HNSCC	Tumor-positive	no		yes	TP
18	HNSCC	Tumor-positive	yes	*n/a*	yes	TP
19	CRC	n/a	*n/a*		*n/a*	*n/a*
20	HNSCC	Tumor-negative	no		yes	FP
21	EC	n/a	*n/a*		*n/a*	*n/a*
22	BC	Tumor-negative	yes	*yes*	yes	FP
23	EC	Tumor-negative	no		no	TN
24	HNSCC	Tumor-negative	no		no	TN
25	EC	Tumor-negative	no		no	TN
26	HNSCC	Tumor-negative	no		no	TN
27	BC	Tumor-positive	no		yes	TP
28	CRC	Tumor-negative	no		no	TN
29	CRC	n/a	*n/a*		*n/a*	*n/a*
30	BC	Tumor-negative (DCIS)	yes	*n/a*	yes	FP?

Supplementary Table 2 | Correlating fluorescence with histopathology per patient

Intraoperative assessment of the surgical margin during fluorescence-guided surgery, can be either done by intraoperative fluorescence imaging of the surgical cavity or fluorescence imaging of the excised specimen at the *'Back-Table'* which is then correlated with the histopathology results on a per patient basis. The surgical resection margins are displayed

according to the US Guidelines per tumor type. Regarding patient 11, no fluorescence imaging of the surgical cavity was performed due to logistics. Regarding patient 18, no biopsy was performed due to the severity of the remaining fluorescence. Regarding patient 30, no biopsy was performed due to logistics. Regarding patients 19, 29 and 21, no tumor containing specimens were excised since peritoneal metastases or a complete response after neoadjuvant treatment was observed respectively.

Abbreviations: BC: Breast cancer; HNSCC: Head and neck squamous cell cancer; EC: Esophageal cancer; CRC: Colorectal cancer; DCIS; Ductal Carcinoma in Situ; TN: True negative; TP: True positive; FP: False positive.
