# Supplementary material for: Exploiting metabolic acidosis in solid cancers using a tumor-agnostic pH-activatable nanoprobe for fluorescence-guided surgery
Source: Nat Commun. 2020 Jun 26;11:3257. doi: 10.1038/s41467-020-16814-4 (PMC7320194; doi:10.1038/s41467-020-16814-4)
Supplement: Supplementary file 3 — Description of Additional Supplementary Files [file 41467_2020_16814_MOESM3_ESM.pdf]

Description of Additional Supplementary Files

File Name: Supplementary Movie 1
Description: Representative video of real-time fluorescence in vivo. A head and neck squamous cell carcinoma of the tongue is shown. A large necrotic ulcer is visible in the middle of the tumor.

File Name: Supplementary Movie 2
Description: Representative video of real-time fluorescence in vivo. A fluorescent signal on a breast cancer specimen is shown during a lumpectomy, when the specimen is still in situ. The fluorescent spot on the specimen correlates with a tumor positive surgical margin (see also Fig. 4). The nipple tissue is emitting high intrinsic autofluorescent signals.

File Name: Supplementary Movie 3
Description: Representative video of real-time fluorescence in vivo. A peritoneal metastasis, originating from a colorectal cancer, is visible on the abdominal wall. The large extraluminal colon tumor mass at the bottom of the screen is moving synchroneous with in- and exhaling during anesthesia.

File Name: Supplementary Movie 4
Description: Representative video of real-time fluorescence in vivo. A head and neck squamous cell carcinoma of the tongue is shown.
