# Supplementary material for: Exploiting metabolic acidosis in solid cancers using a tumor-agnostic pH-activatable nanoprobe for fluorescence-guided surgery
Source: Nat Commun. 2020 Jun 26;11:3257. doi: 10.1038/s41467-020-16814-4 (PMC7320194; doi:10.1038/s41467-020-16814-4)
Supplement: Supplementary file 8 — Reporting Summary [file 41467_2020_16814_MOESM8_ESM.pdf]

natureresearch

Corresponding author(s): B.D. Sumer / G.M. van Dam

Last updated by author(s): May 11, 2020

Reporting Summary

Nature Research wishes to improve the reproducibility of the work that we publish. This form provides structure for consistency and transparency in reporting. For further information on Nature Research policies, see Authors & Referees and the Editorial Policy Checklist .

Statistics

For all statistical analyses, confirm that the following items are present in the figure legend, table legend, main text, or Methods section.

n/a	Confirmed	
☐	☒	The exact sample size (*n*) for each experimental group/condition, given as a discrete number and unit of measurement
☐	☒	A statement on whether measurements were taken from distinct samples or whether the same sample was measured repeatedly
☐	☒	The statistical test(s) used AND whether they are one- or two-sided<br>*Only common tests should be described solely by name; describe more complex techniques in the Methods section.*
☐	☒	A description of all covariates tested
☐	☒	A description of any assumptions or corrections, such as tests of normality and adjustment for multiple comparisons
☐	☒	A full description of the statistical parameters including central tendency (e.g. means) or other basic estimates (e.g. regression coefficient) AND variation (e.g. standard deviation) or associated estimates of uncertainty (e.g. confidence intervals)
☐	☒	For null hypothesis testing, the test statistic (e.g. *F*, *t*, *r*) with confidence intervals, effect sizes, degrees of freedom and *P* value noted<br>*Give P values as exact values whenever suitable.*
☒	☐	For Bayesian analysis, information on the choice of priors and Markov chain Monte Carlo settings
☒	☐	For hierarchical and complex designs, identification of the appropriate level for tests and full reporting of outcomes
☒	☐	Estimates of effect sizes (e.g. Cohen's *d*, Pearson's *r*), indicating how they were calculated

Our web collection on statistics for biologists contains articles on many of the points above.

Software and code

Policy information about availability of computer code

Data collection	In vivo measurements:<br>Two open-surgery intraoperative fluorescent camera systems were used in this study to detect ONM-100, namely the Explorer Air® (SurgVision B.V., Groningen, The Netherlands) and the SPY Elite® (Stryker, Kalamazoo, MI, USA). When minimally invasive surgery was performed (e.g. robot assisted esophagectomy or a diagnostic laparoscopic surgery for peritoneal metastasis), clinically available near-infrared (NIR) imaging systems were used, which are the Olympus NIR Laparoscope (Olympus, Sjinjuku, Tokyo, Japan) and the Intuitive Da Vinci Firefly robot NIR laparoscope (Intuitive Surgical, Sunnyvale, CA, USA)<br><br>Ex vivo measurements:<br>The closed-field macroscopic fluorescence PEARL-trilogy® imaging device (Li-COR BioSciences Inc., Lincoln, NE, USA) was used for whole specimen and tissue slice imaging and is designed for ex vivo fluorescence imaging.<br><br>Pharmacokinetics:<br>Blood was drawn before ONM-100 administration and after 10min, 30min, 1h, 3h, 8h, 24h, 48h, 72h and 240h.<br><br>Safety:<br>Safety assessments were performed before and after ONM-100 administration up to day 17.
Data analysis	Mean Fluorescence Intensity measurements:<br>Image J Fiji (version 2.0.0)<br><br>Statistical analysis:<br>GraphPad Prism, version 8.

Pharmacokinetics:
The analyte ONM-100 was quantified in human plasma (K2EDTA) by fluorescence detection after dilution in phosphate-buffered saline. Calculations: Phoenix WinNonlin (version 8.0).

For manuscripts utilizing custom algorithms or software that are central to the research but not yet described in published literature, software must be made available to editors/reviewers. We strongly encourage code deposition in a community repository (e.g. GitHub). See the Nature Research guidelines for submitting code & software for further information.

Data

Policy information about availability of data

All manuscripts must include a data availability statement. This statement should provide the following information, where applicable:

- Accession codes, unique identifiers, or web links for publicly available datasets
- A list of figures that have associated raw data
- A description of any restrictions on data availability

All the data (imaging data, safety data and pharmacokinetic data) gathered and/or processed during this study are available from the corresponding author on request. The source data (individual data points) underlying Figures 3 and 5, Table 1 and Supplementary Figures 1, 2, 4 and 6 are provided as a Source Data file.

Field-specific reporting

Please select the one below that is the best fit for your research. If you are not sure, read the appropriate sections before making your selection.

[x] Life sciences [ ] Behavioural & social sciences [ ] Ecological, evolutionary & environmental sciences

For a reference copy of the document with all sections, see nature.com/documents/nr-reporting-summary-flat.pdf

Life sciences study design

All studies must disclose on these points even when the disclosure is negative.

Sample size

By previous experience, it has been shown that fluorescence guided surgery dose-escalation studies can be performed by cohorts of three subjects per dose. The first part of the study consisted of 5x3 patients (N=15). To confirm the most optimal dose, we have expanded the sample with an additional 15 patients.

Data exclusions

Pharmacokinetcis: Missing samples (no collection possible due to logistic/clinical situation) were removed from analysis.
Imaging data: For some subjects no whole specimen imaging was performed due to the size of the specimen (did not fit in the PEARL trilogy), this did not affect calculations, since these are based on tissue slides.
Exclusion criteria patients:
Any subjects receiving neoadjuvant therapy prior to surgery were excluded from Phase 1a (to exclude the potential influence of radiotherapy on fluroescence activation in the first 15 subjects). Other exclusion criteria were: an inability to give informed consent; participation in another clinical trial with an investigational product; inadequately controlled hypertension; history of allergic or infusion reaction to iodine, iodine-based contrast, shellfish or ICG; those receiving potentially highly hepatotoxic medication; pregnancy; and subjects with magnesium, potassium and calcium lower than the lower normal limit (for safety reasons).

Replication

Laboratory tests were succesfully performed in triplicates (pharmacokinetcs and in vitro pH activation experiment).

Randomization

Randomization was not performed in this trial since this was a first in human safety, pharmacokinetics and imaging feasibility study.

Blinding

Pathologist were blinded for fluorescence imaging results when performing histopathological analyses.
For pharmacokinetics experiments, analysis was performed blinded.
For TBR calculations, blinding was not possible since H/E sections and fluorescence images need to be correlated/overlayed manually.

Reporting for specific materials, systems and methods

We require information from authors about some types of materials, experimental systems and methods used in many studies. Here, indicate whether each material, system or method listed is relevant to your study. If you are not sure if a list item applies to your research, read the appropriate section before selecting a response.

Materials & experimental systems

n/a	Involved in the study
☒	☐ Antibodies
☒	☐ Eukaryotic cell lines
☒	☐ Palaeontology
☒	☐ Animals and other organisms
☐	☒ Human research participants
☐	☒ Clinical data

Methods

n/a	Involved in the study
☒	☐ ChIP-seq
☒	☐ Flow cytometry
☒	☐ MRI-based neuroimaging

Human research participants

Policy information about studies involving human research participants

Population characteristics	Subjects with histologically proven BC, HNSCC, EC or CRC scheduled for surgical excision were included in this clinical trial (8 males, 22 females). Mean age 63 (range 35-85). All caucasian.
Recruitment	All subjects were identified in the respective multi-disciplinary tumor boards of the participating hospitals. After pre-screening, eligible subjects were orally informed and received written information about the study. After reasonable time to decide for participation, all participants gave written informed consent before the start of any related study procedure.<br>Surgeons were aware of the fluorescence trial, however, they did not perform or were involved in any of the data analysis. Therefore, it is justified to state that chance of selection bias is negligible.
Ethics oversight	Investigational Review Board UMCG (METc Number 2017/580), National Competent Authority (CCMO, ABR NL63129.042.17)

Note that full information on the approval of the study protocol must also be provided in the manuscript.

Clinical data

Policy information about clinical studies

All manuscripts should comply with the ICMJEguidelines for publication of clinical research and a completedCONSORT checklist must be included with all submissions.

Clinical trial registration	The Netherlands National Trial Register (NTR number 7085)
Study protocol	Not online available, the study protocol is available from the corresponding author upon reasonable request. This is by request of the sponsor (OncoNano Medicine Inc.).
Data collection	Subjects signed informed consent and went into screening. If eligible, day 0 of the study was the day of tracer administration nursing ward). Subjects were followed up to day 17 for adverse events monitoring, pharmacokinetic sampling, vital signs and ECG (postoperative on nursing ward and at outpatient clinic).<br>Imaging data was obtained in vivo during surgery (at the operation room) and ex vivo of the excised specimen in the following days (0-10 days) (at the pathology department). Inclusion was open from March 2018 until December 2019.
Outcomes	Primary outcomes were safety, pharmacokinetics and imaging feasibility of ONM-100. Secondary outcomes were to determine a range of safe doses of ONM-100 for intra-operative imaging using commonly used fluorescence camera systems.<br><br>Primary: Subjects were followed up to day 17 for adverse events monitoring, pharmacokinetic sampling, vital signs and ECG. All observed adverse events were reported and judged by the investigators, principal investigator and an independent medical monitor for relation to ONM-100. Fluorescence imaging and correlation with standard histopathology. All in vivo and ex vivo imaging data were analyzed and correlated with histopathological outcome. Images of both sides of the respective tissue slices collected with PEARL-trilogy were used to calculate the Mean Fluorescence Intensity (MFI) of both tumor and background areas. The MFI was based on a Region of Interest (ROI) which was carefully determined on H/E 4 um slides by a board-certified pathologist blinded for fluorescence imaging data and subsequently precise overlayed on the corresponding tissue slice for both tumor and normal tissue. The Tumor-to-Background-Ratio (TBR) was calculated as Tumor ROI (MFI tumor) / background ROI (MFI non-tumor tissue) per tissue slice. Median TBR was calculated on a per subject base. Data (MFO, TBR) was plotted in graphs using GraphPad Prism, version 8.<br><br>Secondary: Dose-escalating carefully monitoring of dose slimiting toxicities. Depending on the tumor type, a variety of intra-operative fluorescence camera's was used.
